# Complications 8 weeks after an obstetric second-degree perineal laceration in relation to body mass index

**DOI:** 10.1007/s00192-023-05609-y

**Published:** 2023-08-16

**Authors:** Maria Otterheim, Linda Hjertberg, Sofia Pihl, Eva Uustal, Marie Blomberg

**Affiliations:** 1 Department of Obstetrics and Gynaecology, Vrinnevi Hospital, Norrköping, Sweden; 2 Department of Obstetrics and Gynaecology, University Hospital, 587 50 Linköping, Sweden; 3 Department of Biomedical and Clinical Sciences, Linköping University, Linköping, Sweden

**Keywords:** Anal incontinence, Body mass index, Obstetric second-degree perineal laceration, Patient-reported outcome, Urinary incontinence

## Abstract

**Introduction and hypothesis:**

How body mass index (BMI) affects pelvic floor function after a second-degree perineal laceration is unknown. The hypothesis of this study is that pelvic floor dysfunction and complications after an obstetric second-degree perineal laceration are more common in women with a higher BMI 8 weeks postpartum.

**Methods:**

This register-based cohort study includes 10,876 primiparous women with an obstetric second-degree perineal laceration between 2014 and 2021. Data were retrieved from the Swedish Perineal Laceration Registry. Outcomes in relation to maternal BMI were urinary incontinence (UI), anal incontinence (AI) and common complications attributable to the laceration. Uni- and multivariate logistic regressions were used for comparison between normal weight (BMI < 24.9, reference), overweight (25.0–29.9) and obese (≥ 30) women.

**Results:**

Multivariate analyses showed an increased risk for UI in both overweight and obese women compared to normal-weight women 8 weeks after a second-degree perineal laceration with an adjusted odds ratio (aOR) of 1.21 (CI 1.02–1.44) and 1.27 (CI 1.13–1.58) respectively. Overweight and obese women had a decreased risk for AI (aOR 0.81, CI 0.68–0.96; aOR 0.72, CI 0.57–0.90 respectively) compared with normal-weight women. No significant differences were found in the univariate analyses over BMI strata concerning complications after perineal laceration.

**Conclusions:**

Primiparous overweight and obese women report less AI and more UI than normal-weight women 8 weeks after a second-degree perineal laceration. No differences were found regarding complications. These findings are new and merit further study to find potential preventive factors and interventions after a second-degree perineal laceration.

**Supplementary information:**

The online version contains supplementary material available at 10.1007/s00192-023-05609-y.

## Introduction

A perineal laceration during childbirth can occur spontaneously or iatrogenically by episiotomy. First-time mothers are more likely to have more extensive perineal lacerations from giving vaginal birth than second-time mothers with a previous vaginal birth [1]. In different studies from around the globe the reported prevalence of perineal lacerations involving the muscles of the pelvic floor varies between 35 and 78% in primiparous women [2–4]. The variations in prevalence are probably due to differences in the diagnostics and definition of perineal tears. The most commonly used classification of obstetric perineal lacerations, which has been adopted by the Royal College of Obstetricians and Gynaecologists and is also used in this study, is the Sultan classification. It defines a second-degree perineal laceration as engaging the muscles in the perineal body, but not the anal sphincter complex [5].

Vaginal birth is a well-known risk factor for pelvic floor dysfunction, including urinary and anal incontinence. The extent of the perineal tear correlates with the severity of pelvic floor dysfunction and complications [6].

Although second-degree perineal tears are more common than third- or fourth-degree tears, they have been less well studied with regard to pelvic floor dysfunction and complications. The postpartum duration and extent of pain from perineal lacerations seem to increase with the severity of the laceration within the first 6 weeks after delivery [7]. A study by Andrews et al. showed that significantly more women experience perineal pain after a second-degree perineal laceration or an episiotomy compared with women with a superficial perineal laceration only involving the vaginal mucosa or with an intact perineum [8]. Furthermore, women are more likely to experience dyspareunia during the first 6 to 12 months postpartum after having a second-degree perineal laceration than women with an intact perineum after a first-time vaginal delivery [9]. The literature is limited concerning urinary incontinence after a second-degree perineal laceration, but perineal lacerations overall seem to increase the risk of urinary incontinence (UI) postpartum [10]. Little is known about anal incontinence (AI) specifically after a second-degree perineal laceration. The Swedish Agency for Health Technology Assessment and Assessment of Social Services (SBU) wrote in a report in 2021, concerning perineal lacerations caused by vaginal birth, that complications that can derive from a perineal laceration include “pain, incontinence, stool issues, psychological suffering, prolapse and sexual difficulties” [11]. According to a meta-analysis consisting of eight studies, episiotomy was associated with a higher risk of AI than no episiotomy, regardless of whether or not the perineal laceration extended into the anal sphincter complex [12].

The share of pregnant women who are classified as overweight or obese in Sweden has increased dramatically [13]. In 2021, 26% of the women in early pregnancy were overweight and 14% were obese while expecting their first child [14]. This pattern is also seen globally, where approximately one third of the general population are overweight or obese [15].

Obesity overall is associated with pelvic floor dysfunction unrelated to pregnancy and vaginal delivery [16]. UI is known to correlate with higher body mass index (BMI) in older age groups after vaginal deliveries, regardless of whether or not a perineal laceration has occurred [17]. It has been indicated that obesity is a risk factor for developing anal incontinence (AI) after a vaginal delivery even if an anal sphincter injury has not been diagnosed [18].

Whether complications related to an obstetric second-degree perineal laceration are affected by maternal overweight and/or obesity has, to our knowledge, not been previously investigated.

The primary aim of this study was to examine the impact of maternal BMI on patient-reported symptoms of pelvic floor dysfunction and complications from an obstetric second-degree perineal laceration 8 weeks after a first vaginal delivery. The outcome measurements included UI, AI, urinary retention, infections, re-operations, visits to an out-patient unit, wound complications and physician-assessed complications. We hypothesised that being overweight and obese increase the risk for UI and AI, at 8 weeks postpartum, and that complications such as urinary retention, infections, re-operations, visits to an out-patient unit, wound complications and physician-assessed complications were more common in overweight and obese women 8 weeks postpartum than in normal-weight women.

## Materials and methods

This was a register-based cohort study including 10,876 primiparous women with an obstetric second-degree perineal laceration derived from a vaginal delivery that took place between January 2014 and December 2021. Data were collected from the Swedish Perineal Laceration Registry (PLR), which began in 2014 and is part of the Swedish National Quality Register of Gynaecological surgery [19]. The register has previously been described in detail [20]. Second-degree perineal lacerations have been reported to the register by a growing number of Swedish delivery units; 26 units in total as of 2021 [11, 21]. The PLR gathers data extracted from the women’s medical journal and survey data on patient-reported outcomes and experiences. Patient-reported data are collected by questionnaires distributed during the first days postpartum and follow-up questionnaires after 8 weeks and 1 year. The questionnaires are distributed either digitally as an online form through the Swedish National Healthcare Guide 1177, by email, or by the postal mail service. A reminder is sent out twice after each of the follow-up questionnaires; after 7 to 14 days and after 14–28 days respectively. The questionnaires have been face-, context-, consistency- and content- validated. This study includes analysis of the pre-pregnancy data and data extracted from the 8-week questionnaires (Appendices 1 and 2).

Collected maternal and obstetrical characteristics were age, BMI, prevalence of diabetes mellitus types I and II (DM), prevalence of inflammatory bowel disease (Crohn’s disease or ulcerative colitis), prevalence of pre-pregnancy UI and AI, foetal presentation at birth, duration of the second stage of labour, episiotomy at delivery, epidural anaesthesia during delivery, mode of vaginal delivery and foetal birth weight. Data on the prevalence of prophylactic antibiotics and pudendal nerve block in connection with repair of the second-degree perineal lacerations were also extracted. BMI was registered in the women’s antenatal care medical record during the first trimester, where it was calculated from the women’s reported height and measured weight.

For analysis concerning maternal and obstetrical characteristics, the study population was divided into five groups according to BMI classes, as suggested by the World Health Organization (WHO): underweight (< 18.5 kg/m^2^), normal weight (18.5–24.9 kg/m^2^), overweight (25.0–29.9 kg/m^2^), obesity (30–34.9 kg/m^2^) and morbid obesity (≥ 35 kg/m^2^) [22]. Maternal and obstetrical characteristics are presented over the BMI strata (Table 1).

**Table 1:** Maternal and obstetrical characteristics in relation to maternal body mass index (*BMI*)

	Total *N* = 10,876	BMI < 18.5, *n* = 292, (2.9%)	BMI 18.5–24.9, *n* = 6,282 (57.8%)	BMI 25.0–29.9, *n* = 2,814 (25.9%)	BMI 30–34.9, *n* = 1,039 (9.6%)	BMI ≥ 35, *n* = 449 (4.1%)	*p* value
Age, mean, years (SD)		28.2 (4.9)	29.4 (4.3)	29.3 (4.4)	29.1 (4.7)	28.64 (4.3)	
Diabetes mellitus, *n* (%)	Yes	2 (0.7)	27 (0.4)	19 (0.7)	11 (1.1)	9 (2.0)	< 0.05
Inflammatory bowel disease, *n* (%)	Yes	1 (0.3)	32 (0.5)	21 (0.7)	3 (0.3)	1 (0.2)	0.28
Pre-pregnancy urinary incontinence, *n* (%)	Yes	3 (1.0)	54 (0.9)	31 (1.1)	15 (1.4)	14 (3.1)	< 0.05
Pre-pregnancy gas and/or faecal incontinence, *n* (%)	Yes	7 (2.4)	180 (2.9)	76 (2.7)	25 (2.4)	15 (3.3)	0.93
Duration of second stage of labour in min, median (IQR)		40.5 (32)	38.0 (31)	35.0 (30)	31.0 (31)	28.0 (30)	< 0.05
Foetal presentation at birth, *n* (%)	Occiput anterior	285 (97.6)	5,989 (95.3)	2,668 (94.8)	9,893 (95.6)	427 (95.1)	
	Occiput posterior	7 (2.4)	228 (3.6)	118 (4.2)	33 (3.2)	19 (4.2)	
	Breech or foot	0	20 (0.3)	7 (0.2)	2 (0.2)	0	
	Other	0	40 (0.6)	18 (0.6)	9 (0.9)	2 (0.4)	
	Unknown	0	5 (0.08)	3 (0.11)	2 (0.19)	1 (0.22)	
Episiotomy, *n* (%)	Yes	80 (27.4)	1,144 (18.2)	399 (14.2)	148 (14.2)	48 (10.7)	< 0.05
Mode of delivery^a^, *n* (%)	Spontaneous vaginal	247 (84.6)	5,033 (80.1)	2,346 (83.4)	891 (85.8)	388 (86.4)	< 0.05
	Instrumental vaginal	45 (15.4)	1,245 (19.9)	463 (16.5)	146 (14.1)	61 (13.6)	
Epidural anaesthesia during labour, *n* (%)	Yes	181 (62.0)	3,950 (62.9)	1,807 (64.2)	711 (68.4)	311 (69.3)	< 0.05
Pudendal nerve block before suturing, *n* (%)	Yes	102 (34.9)	2,201 (35.0)	1,055 (37.5)	371 (35.7)	163 (36.3)	0.26
Prophylactic antibiotics during suturing, *n* (%)	Yes	5 (1.7)	225 (3.6)	100 (3.6)	54 (5.2)	27 (6.0)	< 0.05

*SD* standard deviation, *IQR* interquartile range

^a^The total count of women for mode of delivery does not add up owing to one missing value and ten women who underwent caesarean section. It is considered possible to be diagnosed with a second-degree perineal laceration after a caesarean section if the delivery mode is converted from vaginal to caesarean late during the second stage of labour

The patient-reported outcomes measured by the present study were symptoms of pelvic floor dysfunction, defined in the current study as the prevalence of UI (defined as urinary leakage once a week or more), urinary retention (yes/no) and AI (defined as any reported involuntary loss of faeces or flatus; yes/no) as well as complications of the laceration, defined as any infections treated with antibiotics, re-operation (yes/no), unplanned visits to an out-patient unit (yes/no), wound complications (yes/no) and “Other complications” (further assessed by a physician as none, mild or severe).

The group of women who reported any anal incontinence in the questionnaire were presented with further questions that were used to compose the Wexner score for anal incontinence [23]. The supplementary questions requested information regarding incontinence of gas, liquid stool, solid stool, use of pads, change of lifestyle and how often these parameters presented (never/rarely/1–3 times monthly/1–2 times weekly/daily). These parameters of the Wexner score were dichotomised into “yes” or “no” with the cut-off for yes at once a week or more. The Wexner score has previously been used with a cut-off level of ≥ 2 to define a degree of anal incontinence that affects quality of life after an obstetric anal sphincter injury (OASI) [23]. We used the same cut-off level when dichotomising the total Wexner score in relation to second-degree lacerations. We have no reason to believe that the cut-off level for the Wexner score in relation to quality of life would differ between the types of laceration.

Patient-reported outcome measures (PROM) were analysed in relation to BMI divided into three groups; normal weight ≤ 24.9 kg/m^2^, overweight 25–29.9 kg/m^2^ and obese ≥ 30 kg/m^2^. The women with BMI ≤ 24.9 kg/m^2^ were set as the reference group and are hereafter referred to as being of normal weight.

### Ethical approval

The Regional Ethical Review Board in Linköping approved the study on 20 April 2016; Dnr 2016/144-31.

### Statistics

All data analyses were performed using SPSS version 27 (IBM, Armonk, NY, USA). Categorical background data were presented as total number of patients (*N*) and percentage of each variable in each BMI group in the descriptive analyses. Continuous data were presented as mean and standard deviation (SD) or median and interquartile range (IQR), if not normally distributed. Categorical variables concerning maternal and obstetrical characteristics were analysed using Pearson’s Chi-squared test. The mean value of age was compared using analysis of variance (ANOVA). The duration of the second stage of labour and days to resumed activities of daily living (ADL) in the numerical background data were analysed using non-parametric tests. *p* values < 0.05 were regarded as significant.

The three BMI groups were compared using binary logistic regression analyses. Risk estimates were presented as crude odds ratios (ORs) with 95% confidence intervals (CIs).

Further comparisons among the BMI groups were made using multivariate analysis. Where significant differences were found, the results were adjusted for the proven confounders. Univariate analysis was used to select potential confounders. The variables with a significant relation to the outcome in the primary multivariate analysis were included in the final calculation of the adjusted odds ratio (aOR), presented with a 95% CI.

## Results

Urinary incontinence was significantly more frequently reported among overweight and obese women at 8 weeks postpartum (Table 2). The absolute rates of UI 8 weeks postpartum were 12.2% (*n* = 587) among normal weight women, 14.4% (*n* = 296) among overweight women and 15.5% (*n* = 169) in obese women. Overweight women with a second-degree perineal laceration had a 21% increased risk for reported UI (OR 1.21; 95% CI 1.21–1.41) compared with normal-weight women, and the corresponding risk for obese women was 32% (OR 1.32 CI 1.10–1.59) at 8 weeks postpartum. The altered risk for UI remained significant when adjusted for age, pre-pregnancy UI and pre-pregnancy AI in both overweight women (aOR 1.21, CI 1.02–1.44) and obese women (aOR 1.27; 95% CI 1.13–1.58; Table 3). The overweight and obese women reported less AI than normal-weight women 8 weeks after a second-degree perineal laceration (Table 2). The absolute rate of reported incontinence for gas or faeces was 15.9% (*n* = 753) in normal-weight women, 13.6% (*n* = 276) in overweight women and 11.8% (*n* = 127) in obese women. Overweight women had a 17% decreased risk for AI (OR 0.83 CI; 0.72–0.96) and obese women had a 29% decreased risk (OR 0.71; CI 0.58–0.86), compared with normal-weight women. When adjusted for age, pre-pregnancy AI, duration of second stage of labour, episiotomy and mode of delivery, the decreased risk of self-reported AI remained significant for both overweight women (aOR 0.81; 95% CI 0.68–0.96) and obese women (aOR 0.72; 95% CI 0.57–0.90) compared with normal-weight women (Table 3).

**Table 2:** Patient-reported outcome measures data at 8 weeks postpartum after a second-degree laceration in relation to maternal body mass index (*BMI*)

	BMI ≤ 24.9		BMI 25.0–29.9			BMI > 30		
	*n* (%)	OR	*n* (%)	OR	95% CI	*n* (%)	OR	95% CI
Reported urinary incontinence	587/4,826 (12.2)	1.00	296/2,058 (14.4)	**1.21**	1.04–1.41	169/1,091 (15.5)	**1.32**	1.10–1.59
Reported urinary retention	61/5,016 (1.2)	1.00	30/2,150 (1.4)	1.15	0.74–1.79	14/1,128 (1.2)	1.02	0.57–1.83
Reported incontinence for gas or faeces	753/4,739 (15.9)	1.00	276/2,036 (13.6)	*0.83*	0.72–0.96	127/1,078 (11.8)	**0.71**	0.58–0.86
Reported gas incontinence^a^	411/749 (54.9)	1.00	148/276 (53.6)	0.95	0.72–1.25	60/126 (47.6)	0.75	0.51–1.09
Reported incontinence for loose stool^a^	44/747 (5.9)	1.00	8/274 (2.9)	0.48	0.22–1.03	12/125 (9.6)	1.70	0.87–3.31
Reported incontinence for solid stool^a^	31/749 (4.1)	1.00	5/273 (5.0)	0.43	0.17–1.12	6/125 (4.8)	1.17	0.48–2.86
Reported usage of pads for faecal incontinence^a^	35/750 (4.7)	1.00	12/275 (4.4)	0.93	0.48–1.82	7/125 (5.6)	1.21	0.53–2.80
Change of lifestyle due to faecal incontinence^a^	79/749 (10.5)	1.00	31/274 (11.3)	1.01	0.70–1.68	20/125	1.62	0.95–2.75
Wexner score ≥ 2 (^a^ included variables)	136/4,916 (2.8)	1.00	61/2,123 (2.9)	1.04	0.77–1.41	31/1,125 (2.8)	1.00	0.67–1.48
Infections treated with antibiotics	282/5,015 (5.6)	1.00	123/2,148 (5.7)	0.98	0.79–1.22	59/1,128 (5.2)	1.08	0.81–1.44
Reported re-operation	99/5,016 (2.0)	1.00	52/2,150 (2.4)	0.81	0.58–1.14	25/1,128 (2.2)	0.89	0.57–1.38
Reported visit at out-patient unit	694/5,016 (13.8)	1.00	302/2,150 (14.0)	0.81	0.85–1.14	146/1,128 (12.9)	0.43	0.89–1.31
Reported wound complication	502/5,016 (10.0)	1.00	199/2,150 (9.3)	0.92	0.77–1.09	105/1,128 (9.3)	0.92	0.74–1.15
Physician assessment of reported complication	724/4,741 (15.3)	1.00	283/2,030 (13.9)	0.90	0.78–1.04	145/1,068 (13.6)	0.87	0.72–1.06

The number of replies and the percentage are given in each row

Binary logistic regression analyses were used for comparison between the BMI groups

Risk estimates are presented as OR with 95% CIs

Significant ORs are shown in bold figures

*CI* confidence interval, *OR* odds ratio

^a^Questions included in the Wexner score system

**Table 3:** Patient-reported data of urinary and anal incontinence at 8 weeks postpartum after a second-degree laceration in relation to maternal body mass index (*BMI*), adjusted data

	BMI ≤ 24.9		BMI 25.0–29.9			BMI > 30		
	*n* (%)	OR	*n* (%)	OR	95% CI	*n* (%)	OR	95% CI
Reported urinary incontinence^a^	587/4,826 (12.2)	1.00	296/2,058 (14.4)	**1.21**	1.02–1.44	169/1,091 (15.5)	**1.27**	1.13–1.58
Reported incontinence for gas or faeces^b^	753/4,739 (15.9)	1.00	276/2,036 (13.6)	**0.81**	0.68–0.96	127/1,078 (11.8)	**1.27**	0.57–0.90

Significant ORs are shown in bold figures

^a^Adjusted for age, pre-pregnancy urine incontinence and pre-pregnancy incontinence for gas or faeces

^b^Adjusted for age, pre-pregnancy incontinence for gas or faeces, duration of second stage of labour, episiotomy and mode of delivery (spontaneous or instrumental vaginal delivery)

According to the Wexner score system, BMI did not have any impact on the type or influence of anal incontinence in the group of 1,156 women reporting any incontinence for gas or faeces.

The Wexner score has been used with a cut-off level of ≥ 2 to estimate the effect of quality of life in women with AI postpartum [23]. The reported quality of life according to the Wexner score did not differ between the BMI groups.

Regarding complications, the rates of reported infections treated with antibiotics did not differ between the BMI groups. There was a tendency towards fewer self-reported complications such as re-operations, fewer visits to out-patient units and fewer wound complications among the overweight and obese women than among the normal-weight women, but there were no statistically significant differences among groups. The rate of self-reported variable “other complications” was somewhat lower among overweight and obese women than among normal-weight women, but the difference was not statistically significant.

Median reported days to resumed normal ADL was similar, 6 days, across the BMI strata (Table 2).

Maternal and obstetrical background characteristics of the study population (*N* = 10,876) are shown in Table 1. In the study population, 2.9% were underweight, 57.8% were of normal weight, 25.9% were overweight, 9.6% were obese and 4.1% were morbidly obese.

There was a significant difference in the proportion of women with diabetes mellitus between the BMI groups, with a higher ratio of diabetes mellitus with higher BMI. Among the normal-weight women, 0.4% had diabetes mellitus compared with 2.0% of the morbidly obese women. Patient-reported pre-pregnancy urine incontinence was significantly more common in women with a higher BMI. Of the morbidly obese women, 3.1% reported they had been affected by UI, compared with 0.9% of women with normal BMI (*p* < 0.05).

Pre-pregnancy gas and/or faecal incontinence did not differ significantly over the BMI strata. The duration of the second stage of labour decreased with higher BMI, where normal-weight women had a median time of 40.5 min and morbidly obese women had a median time of 28 min (*p* < 0.05). Epidural anaesthesia was more frequently used during labour in groups with higher BMI: 69.3% of the morbidly obese women compared with 62.9% of women with normal BMI (*p* < 0.05). Episiotomy was more common in women with lower BMI, where 27.4% of the underweight women and 18.2% of the normal weight women underwent the procedure, compared with 10.7% of the morbidly obese women (*p* < 0.05).

Among the normal-weight women, 80.1% had a spontaneous vaginal delivery compared with 86.4% of the morbidly obese women (*p* < 0.05). Furthermore, there was also a significant difference in the proportion of women who received prophylactic antibiotics during surgery, where 3.6% of the normal-weight women and 6.0% of the morbidly obese women received prophylactic antibiotics (*p* < 0.05).

The 8-week follow-up questionnaires had a response rate of 73.3%. The proportions of the exposure (BMI) among non-responders were as follows: 60.2% of normal-weight women, 25.8% of overweight women and 14.0% of obese women, i.e. very similar to the study population and thus without clinical relevance.

## Discussion

This study showed that women with overweight or obesity were at a lower risk of developing AI at 8 weeks postpartum than normal-weight women, after a second-degree perineal laceration derived from a first vaginal delivery. This is a new finding that contradicts our stated hypothesis and to our knowledge has not previously been presented. The study also showed that overweight and obese women were at a higher risk of developing UI at 8 weeks postpartum than normal-weight women, which is consistent with our hypothesis. The literature regarding UI and AI in relation to BMI after a second-degree perineal laceration is very limited. Dolan and Hilton have shown that obesity is related to UI in older age groups, independent of obstetric history [17]. That UI correlates with BMI as early as 8 weeks after a second-degree perineal laceration has been indicated before in a qualitative study by Lindberg et al. They suggest that second-degree perineal lacerations might have a connection to UI and AI but are also associated with pain, delayed resumption of sexual life, haemorrhoids and problems with stitches during the first months after childbirth [24].

The reason for the decreased risk of AI among obese women after a second-degree perineal laceration can only be speculated upon. Overweight and obese women in the present study underwent episiotomy less often than normal-weight women. A meta-analysis from 2015 showed that episiotomy increased the risk of anal incontinence, ranging over a follow-up time of 8 weeks to 14 years [12]. In the present study, AI remained significantly more common in normal-weight women than in overweight and obese women, with or without episiotomy.

In this material, obese women have a significantly shorter second stage of labour, which is consistent with the findings of a Swedish historical prospective cohort study [25]. The shorter second stage of labour may be a protective variable to the levator muscles and the pudendal nerve area in overweight and obese women, even when a second-degree perineal laceration occurs. One study has shown that the anovaginal distance is longer in obese women having a vaginal birth than in their normal-weight counterparts, which may be protective for the perineal body complex [26]. That the levator muscles could be less affected in overweight and obese women with a second-degree perineal laceration might contribute to the lower incidence of AI in these groups than in normal-weight women with the same degree of laceration. Future studies are needed to further evaluate these possible explanations.

There are, to the best of our knowledge, no earlier publications about BMI in relation to pelvic floor function after a second-degree perineal laceration. The vast majority of studies made on perineal lacerations have focused on OASIs [27]. Many of them have shown a relationship between an OASI and urinary and/or anal incontinence [12]. A recent study evaluated complications 8 weeks after a first-time vaginal delivery complicated by an OASI, in relation to BMI. Their study population included 4,978 women with an OASI, and the conclusion was that overweight and obese women were at a lower risk of developing AI and a higher risk of developing UI than their normal-weight counterparts at 8 weeks postpartum [28]. Those findings are consistent with the findings of this study.

Hjertberg et al. found a lower risk of wound complications in overweight women than in normal-weight women during the first 8 weeks after an OASI [28]. Gommesen et al., found a risk of wound infection more than seven-fold higher in morbidly obese women (BMI > 35) with any type of perineal laceration during the first 3 weeks postpartum than in normal-weight women [29]. In this study we did not see any difference in wound complications between different BMI groups after a second-degree perineal laceration during the first 8 weeks postpartum. However, there was a significant difference in the number of women receiving antibiotics during surgery, where morbidly obese women received prophylactic antibiotics at a significantly higher rate than normal-weight women. One can speculate that this difference might have affected the outcome of wound complications, although the total number of women receiving prophylactic antibiotics was low.

The main findings of the study could be implemented in targeted patient information postpartum and to tailor care related to these risks. Obese women often receive information with negative connotations throughout their pregnancy. Both the woman and the clinician may find a small but positive counterweight in the finding that obese women are at a decreased risk of AI.

A strength of the present study is that the PLR contains a large cohort of women available for evaluation. The response rate to the questionnaires was high and there was detailed information about all the women who were included. Information concerning pre-pregnancy status, maternal health characteristics, the repair of the second-degree perineal lacerations and the 8-week follow-up questionnaires was included. The detailed PROM data enabled adjusted calculations on confounders to further strengthen the power of the results.

Register studies are generally limited when it comes to coverage and missing values. The questionnaires were only available in Swedish, which is a limitation, as a language barrier is considered to increase the risk of adverse effects related to childbirth.

The PLR does not cover all second-degree perineal lacerations nationally, as not all clinics report this type of laceration. The proportion of reported second-degree lacerations differs between clinics, but there is no reason to believe that this variation in reporting or selection of reporting clinics is related to maternal BMI.

Another shortcoming is that historically, there has not been a consensus on how to define second-degree perineal lacerations. Using the most recognised way of defining perineal lacerations, the Sultan classification, second-degree lacerations can range from having a minor impact on the perineal body to an exposed but unharmed anal sphincter [5]. This naturally makes second-degree perineal lacerations a heterogenic group to study. New diagnosis codes in the International Statistical Classification of Diseases and Related Health Problems, covering subgroups of second-degree perineal laceration, were launched in 2020 on the initiative of the Swedish Association of Obstetricians and Gynaecologists [30]. As this categorisation was implemented during the study period of this study, the Sultan classification has been used throughout the present study. The purpose of the new classification is to better diagnose the extent of each second-degree perineal laceration. This will also make it possible to analyse the subtypes of second-degree perineal lacerations in relation to BMI and hopefully find possible preventive factors.

## Conclusion

This large register study showed that overweight and obese women are at a decreased risk of AI but a higher risk of UI, compared with normal-weight women 8 weeks after a first vaginal delivery complicated by a second-degree perineal laceration. No differences were found between the BMI groups regarding the complication measurements including infections, wound complications, urinary retention, re-operations, visits to out-patient units and physician-assessed complications 8 weeks postpartum. The high incidence of obstetric second-degree perineal lacerations, in combination with growing numbers of overweight and obese women, makes this a crucial field to investigate further in order to offer relevant care and give applicable advice to women postpartum. Potential preventive factors for AI associated with higher BMI, as well as possible interventions to decrease the proportion of women suffering from UI, merit further study.

### Supplementary information

Below is the link to the electronic supplementary material.

Appendix 1 Information about your health prior to pregnancy (PDF 201 KB)

Appendix 2 Your assessment after the repair of the perineal tear (PDF 125 KB)

## Data Availability

Raw data were generated at the PLR. Derived data supporting the findings of this study are available from the corresponding author (MB) on request.

## Abbreviations

ADL: Activities of daily living
AI: Anal incontinence
aOR: Adjusted odds ratio
BMI: Body mass index (kilograms/m^2^)
CI: Confidence interval
IQR: Interquartile range
OASI: Obstetric anal sphincter injury
OR: Odds ratio
PLR: Perineal Laceration Registry
PROM: Patient-reported outcome measure
SD: Standard deviation
UI: Urinary incontinence
